# Parenterally administered pegbovigrastim alters leukocyte counts, granulocyte functions, and uterine cell population in healthy postpartum dairy cows

**DOI:** 10.1371/journal.pone.0342743

**Published:** 2026-02-13

**Authors:** Dinesh Dadarwal, Kira Crooks, Patricia Lainetti, Ryan Dickinson, Khawaja Ashfaque Ahmed, Colin Palmer

**Affiliations:** 1 Large Animal Clinical Sciences, Western College of Veterinary Medicine, University of Saskatchewan, Saskatoon, Canada; 2 Veterinary Pathology, Western College of Veterinary Medicine, University of Saskatchewan, Saskatoon, Canada; INRAE Centre Val de Loire: Institut National de Recherche pour l'Agriculture l'Alimentation et l'Environnement Centre Val de Loire, FRANCE

## Abstract

This study aimed to evaluate the effects of a single postpartum administration of pegbovigrastim, a recombinant bovine granulocyte colony-stimulating factor (rG-CSF), on peripheral leukocyte profiles, granulocyte function, and uterine cytology in healthy Holstein dairy cows. We hypothesized that rG-CSF would enhance leukocyte counts and granulocyte function without adversely affecting uterine immune cell composition. Twenty-three cows between 19–23 days in milk were randomly assigned to receive either rG-CSF (n = 12) or saline (n = 11). Blood samples were collected on the day of injection and on Days 3, 6, 10, and 21 post-treatment to assess total and differential leukocyte counts. Granulocyte phagocytosis of fluorescein isothiocyanate (FITC)-labeled *Staphylococcus aureus* and oxidative burst capacity following PMA stimulation were evaluated using flow cytometry. Vaginoscopy and transrectal ultrasound examinations were conducted at each time point, and uterine cytobrush samples were collected from a subset of cows for cytological analysis. Compared to controls, rG-CSF-treated cows exhibited a significant (2–3 fold) increase in total leukocytes and neutrophils (*P* < 0.01) on Days 3, 6, and 10. Monocyte counts were also elevated (4-fold; *P* < 0.01) on Day 3. Granulocyte functional assays revealed increase in oxidative burst (*P* = 0.04) and phagocytic activity as well as capacity (*P* = 0.01) that peaked on Days 3 and 6 post-treatment, respectively, following rG-CSF treatment. Furthermore, uterine samples from treated cows showed higher proportions of neutrophils (Days 6, 10, and 21) and macrophages (Day 10) compared to controls (*P* < 0.01). In conclusion, a single dose of rG-CSF in early postpartum cows induces transient leukocytosis and enhances granulocyte function. The observed increase in uterine neutrophils and macrophages suggests that rG-CSF could be explored further for its potential as a local immunomodulatory agent during the postpartum period.

## Introduction

Innate immune cells, especially neutrophils, play a significant role in the cleanup and repair (involution) of the postpartum uterus [1,2]. Neutrophil influx into uterine tissue associated with involution is most evident during the first three weeks, which subsides by 35 days in milk (DIM) in normal cows [2,3]. However, in some cases, neutrophil presence persists beyond 29–35 DIM, remaining confined to the mucosal and submucosal layers of the uterus. This condition is classified as endometritis. Affected cows often exhibit increased expression of pro-inflammatory cytokines (IL-1α, IL-1β, IL-8, and TNF-α) as well as anti-inflammatory mediators (IL-10 and IL-1 receptor antagonist) [4], indicating an imbalance between the mucosal immune response and pathogen(s) [1,2]. Although clinical signs of endometritis may be subtle or absent, it is consistently associated with reduced reproductive performance [1]. Traditional treatments for endometritis include prostaglandin F2α protocols [5,6], which promote estrus induction, and intrauterine or parenteral antibiotics [7]. While antimicrobials are typically more effective, prostaglandin protocols offer a more physiological approach without contributing to antimicrobial resistance in food-producing animals. The need to preserve reproductive efficiency amid growing concerns over antimicrobial use has stimulated interest in harnessing innate immune mechanisms for alternative therapies.

Granulocyte colony-stimulating factor (G-CSF, or CSF-3) is a cytokine that stimulates bone marrow to release myeloid progenitor cells into circulation, promoting their differentiation into neutrophils [8]. Beyond inducing neutrophilia, G-CSF also enhances neutrophil function, including phagocytosis, and is used clinically in neutropenic patients undergoing chemotherapy [8]. After initial trials in the early 90s, research carried out during the last decade has reported that recombinant (r)G-CSF is helpful as an alternative to antibiotics in reducing the incidence of mastitis in dairy cattle [9]. For this, treatment is typically started a week before calving and then repeated within 24–48 h following calving and has been shown to increase circulating neutrophils and enhance neutrophil phagocytic activity during the early postpartum period [10–13]. While these systemic effects are well-documented, the extent to which this expanded peripheral leukocyte pool traffics to the uterine mucosa remains unknown. More recent clinical trials have shown the benefits of rG-CSF on improved economic returns mainly due to reduced culling rate in dairy herds [12,14]. While previous reports have demonstrated that an inadequate early neutrophil response contributes to (sub)clinical endometritis, it remains unclear whether simply increasing neutrophil numbers earlier fully resolves this immune dampening [15–17]. Moreover, rG-CSF treatment during the immediate postpartum period may coincide with transient physiological immunosuppression, reducing therapeutic efficacy [18]. Thus, we hypothesized that treatment later in the postpartum period, when neutrophil function begins to recover but uterine clearance is still incomplete, may offer a therapeutic window to boost uterine immunity more effectively. An important aspect to consider with rG-CSF therapy is that there is no meat or milk withdrawal due to the absence of residues, and the milk content is unaltered [19]. It is important to note that although pegbovigrastim (rG-CSF) was commercially available at the time of study design, it has since been withdrawn from the market, primarily due to limited uptake driven by cost concerns.

In postpartum cows, neutrophils dominate the uterine cytology (~68% of total cells obtained on endometrial cytology) during the first week in response to bacterial contamination [2,3]. By the fourth week, neutrophils decline to ~15%, coinciding with bacterial clearance and near completion of uterine involution [2,3]. This natural decline in local immune presence occurs precisely when the uterus may still harbor residual pathogens, suggesting that rG-CSF treatment during the third or fifth week postpartum might be advantageous by artificially sustaining the leukocyte population. Furthermore, previous research has shown that rG-CSF not only causes neutrophilia but also increases circulating lymphocytes and monocytes (precursors to macrophages) [20]. However, it remains unclear whether elevated peripheral leukocytes lead to a corresponding increase in the migration of immune cells into the uterus, particularly if rG-CSF treatment occurs during the third week postpartum.

This study is part of a broader research effort to evaluate immunomodulatory therapies for the prevention and treatment of uterine inflammation. Such approaches are essential for reducing antimicrobial resistance, ensuring safe milk and meat production, and improving the economic sustainability of the dairy and beef industries. To explore the connection between systemic stimulation and local uterine response, we hypothesized that parenteral administration of pegbovigrastim (rG-CSF), a non-antibiotic immunomodulator, would enhance the phagocytic and oxidative burst capabilities of granulocytes in addition to inducing neutrophilia. We also tested the null hypothesis that rG-CSF treatment would not affect the proportions of uterine leukocytes, particularly neutrophils and macrophages, in healthy postpartum cows. Therefore, the objective of this study was to evaluate the effects of rG-CSF on peripheral leukocyte counts, granulocyte function (phagocytosis and oxidative burst), and uterine immune cell populations in healthy postpartum dairy cows.

## Materials and methods

### Management of experiment animals

All animal procedures for this study were conducted at the Rayner Dairy Research and Teaching Facility, University of Saskatchewan, between May 2018 and June 2019. The facility housed a herd of approximately 115 lactating Canadian Holstein cows, with an average daily milk production of 43 kg per cow. Cows were maintained in group-housed pens under standard management and feeding conditions throughout the study. All experimental protocols were reviewed and approved by the University of Saskatchewan’s Animal Research Ethics Board (Animal Use Protocol #20180020) and complied with the guidelines set forth by the Canadian Council on Animal Care for the ethical use of animals in research.

### Animal recruitment and treatment

Postpartum cows without an episode of periparturient metabolic (e.g., hypocalcemia, ketosis, displaced abomasum) or reproductive (e.g., retained placenta, metritis) diseases were identified using computerized health records (DairyComp 305). Additionally, cows with abnormal calvings, including dystocia, twins, abortion, stillbirth, or malpresented calves, were excluded to avoid confounding effects on postpartum immune function. Only clinically healthy cows with an unassisted, singleton calving were eligible for enrollment. Due to strict inclusion criteria and concurrent research activity in the herd, cows already participating in other trials were excluded from this study. As a result, enrollment occurred over an extended period (>1 year) to ensure adequate sample size without compromising the experimental controls.

**Fig 1:**
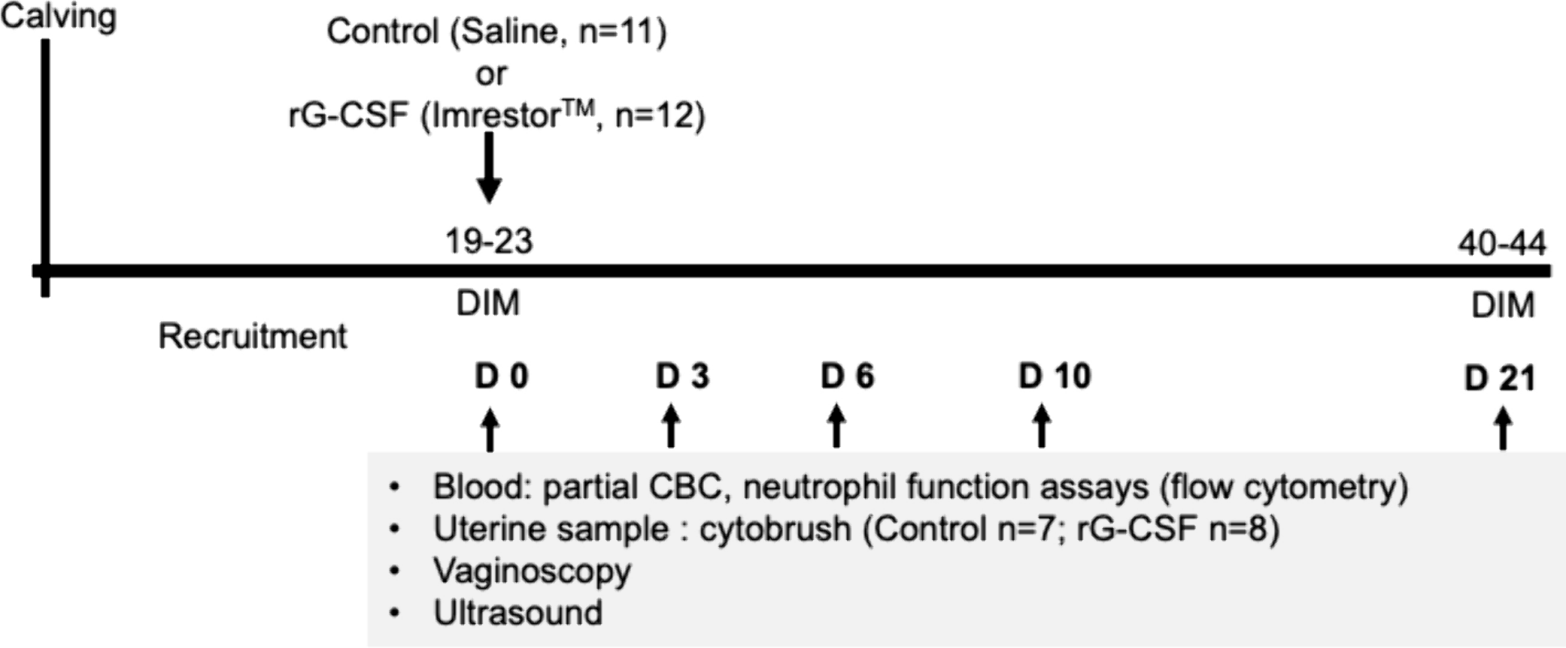
Experimental design of the clinical trial. Postpartum dairy cows (19–23 DIM) were randomly assigned to receive either pegbovigrastim (rG-CSF; recombinant granulocyte colony-stimulating factor) or saline (Control). Cows were examined and sampled immediately prior to treatment (Day 0) and subsequently on Days 3, 6, 10, and 21 post-treatment. Uterine cytobrush samples from a subset of cows were collected on Days 0, 3, 6, 10, and 21. Clinical examinations, including vaginoscopy to assess vaginal discharge and transrectal ultrasonography to detect intrauterine fluid accumulation, were performed to diagnose clinical endometritis.

Thirty clinically healthy postpartum cows (19–23 DIM) were initially identified for the study and randomly allocated (accounting for age and parity) to two treatment groups. The animals were administered either: 1) rG-CSF – a single dose of pegbovigrastim (rG-CSF) sc (15 mg, 2.7 ml, Imrestor^TM^, Elanco Canada); or 2) Control – sterile saline (2.7 ml, 0.9% sodium chloride, Hospira, Kirkland, QC, Canada) subcutaneously. On the day of treatment, cows assigned to the rG-CSF group were sampled and treated before cows in the Control group. This allowed us to monitor the cows of the rG-CSF group for any adverse reactions to the pegbovigrastim treatment; none were observed. Seven cows were excluded from the trial due to treatment for an illness unrelated to the treatment (5/7 mastitis or lameness) or culling (2/7) that occurred during the study. As a result, 23 cows remained in the trial (n = 12 in the rG-CSF group, age 45.5 + 26.8 months, parity 2.1 + 1.7; n = 11 in the Control group, age 49.4 + 32.0 months, parity 2.3 + 1.4), and the experimental design is shown in Fig 1.

Before the treatment on Day 0, all cows were examined by vaginoscopy to assess the presence and characteristics of vaginal discharge and transrectal ultrasonography to determine the ovarian activity and evaluate the intra-uterine contents. Additional data on body condition score (BCS), body weight, and average daily milk yield of each animal were collected at the start of the experiment. In the rG-CSF group, the mean BCS was 2.7 ± 0.3 (on a 5-point scale), body weight was 693.8 ± 175.2 kg, and average daily milk yield was 37.5 + 8.4 L/day. In the control group, the corresponding values were a BCS of 2.9 ± 0.3, body weight of 693.8 ± 175.2 kg, and average daily milk yield of 39.1 + 7.4 L/day. Blood samples were taken before the treatment for total and differential leukocyte counts, phagocytosis, and oxidative burst capacity of granulocytes. These examinations and blood sampling were repeated on Days 3, 6, 10, and 21 after the treatment. Additionally, in a subset of recruited animals used for blood collection (Control group: *n* = 7; rG-CSF group: *n* = 8), uterine samples were collected using a cytobrush on each sampling day to assess the dynamics of cytological changes in the uterus.

### Blood collection, total and differential blood cell counts

Blood samples were collected via coccygeal venipuncture using K₂EDTA collection tubes (Cat #367844, BD Vacutainer, Franklin Lakes, NJ, USA) for total and differential leukocyte counts, and lithium heparin tubes (Cat #367880, BD Vacutainer) for flow cytometry analysis. After collection, all tubes were gently inverted to ensure proper mixing of the anticoagulant and blood, then placed in a cooler containing crushed ice for transport. Samples designated for leukocyte enumeration by automated hematology analyzer were submitted to Prairie Diagnostic Services Inc. (Saskatoon, SK, Canada). Blood samples intended for assessment of granulocyte function were processed as described in the subsequent sections.

### Peripheral granulocyte phagocytosis and oxidative burst assays

All samples were processed for peripheral granulocyte function assays within one hour of blood collection. An established method for in vitro assessment of granulocyte phagocytic function in human whole blood was adapted for use in bovine samples [21]. Heparinized whole blood was incubated with fluorescein isothiocyanate (FITC)-labeled *Staphylococcus aureus* (Cat #352058, Fisher Scientific, Ottawa, ON, Canada), and granulocyte-associated green fluorescence was measured using flow cytometry with specific antibodies. Each blood sample was aliquoted (100 µL each) into three polypropylene tubes representing the test sample, isotype control, and negative control. To ensure a standardized challenge to the peripheral immune cells, FITC-labeled *S. aureus* (30 µL, ~ 133,333 bacteria/µL) was added to the test and isotype control tubes to maintain a multiplicity of infection of 8 bacteria per leukocyte. The negative control remained untreated. All tubes were incubated in a 37 °C water bath for 20 minutes with intermittent vortexing (2–3 seconds every 5 minutes) to facilitate phagocytosis. The reaction was stopped by placing the tubes on ice, followed by the addition of 100 µL of quenching solution (750 µL of 0.4% trypan blue in 11.25 mL of 0.1 M citrate buffer, pH 4) to remove non-phagocytosed bacteria. Samples were washed twice with phagocytosis assay buffer (PBS + 1% fetal bovine serum) and centrifuged at 974g for 5 minutes. Red blood cells were lysed by incubating the pellets in 2 mL of RBC lysis solution (10x RBC Lysis Buffer, Cat #420301, BioLegend, diluted 1:10 in Milli-Q water) at 37 °C for 5 minutes. After a 10-minute centrifugation (974g), the pellets were resuspended in 100 µL of phagocytosis assay buffer and kept on ice. To label granulocytes, 50 µL of anti-bovine granulocyte monoclonal antibody (clone CH138A, Cat #WS0609B-100, Kingfisher Biotech, Saint Paul, MN, USA; 1:50 dilution) was added to all tubes except the isotype control and incubated on ice for 15 minutes. After two washes with 400 µL of assay buffer, 100 µL of secondary antibody (Allophycocyanin AffiniPure F(ab’)₂ Fragment Donkey Anti-Mouse IgG, Cat #715-136-151, Jackson ImmunoResearch, PA, USA; 1:100 dilution) was added, followed by a second 15-minute ice incubation. Tubes were washed twice more, resuspended in 200 µL of assay buffer, and stored on ice until flow cytometry.

For the assessment of oxidative burst capacity of granulocytes, we evaluated the conversion of non-fluorescent dihydrorhodamine (DHR) 123 to fluorescent rhodamine 123 using a Neutrophil/Monocyte Respiratory Burst Assay Kit (Cayman Chemical, Ann Arbor, MI, USA) with minor modifications. Briefly, 100 µL of heparinized whole blood was added to each test, isotype, and negative control tube and first incubated with 10 µL of DHR123 (0.5 mg/mL) at 37 °C for 15 minutes to allow for intracellular probe loading. Then, 25 µL of protein kinase C agonist phorbol 12-myristate 13-acetate (PMA, 200 nmol/L) was added to the test and isotype control tubes, to induce the production of reactive oxygen species, but not to the negative control. After a second 15-minute incubation at 37 °C, the tubes were placed on ice. Subsequent steps including hemolysis, antibody labeling, and washes were performed as described for the phagocytosis assay. Finally, pellets were resuspended in 100 µL of oxidative burst assay buffer (RPMI 1640 base medium supplemented with 1 mmol/mL calcium chloride and 10 mg/mL bovine serum albumin) and kept on ice until flow cytometry analysis.

### Flow cytometry

Samples were analyzed using a CytoFLEX Flow Cytometer (Beckman Coulter Canada Inc., Mississauga, ON, Canada), and data were processed using FlowJo v10 software (Tree Star, Inc., Ashland, OR, USA). Prior to analysis, all samples from both phagocytosis and oxidative burst assays were filtered using cell strainer tubes (Cat# 08-777-23, Fisher Scientific, Ottawa, ON, Canada) to remove debris. Leukocyte populations were initially identified based on their forward scatter (FSC) versus side scatter (SSC) characteristics. Granulocytes were subsequently gated using an anti-bovine granulocyte–APC fluorescence versus SSC plot. A minimum of 15,000 granulocytes were acquired per sample. Total leukocyte populations were used to calculate the proportion of granulocytes. Gated granulocytes (APC-labeled) were further analyzed for FITC fluorescence to assess phagocytosis or fluorescent rhodamine 123 to assess oxidative burst. Both the proportion of FITC-positive granulocytes and their median fluorescence intensity (MFI) were recorded. To account for background signal, the MFI values obtained from the corresponding negative control samples were subtracted from those of the test samples. This corrected MFI was used as a quantitative measure of phagocytic or oxidative burst capacity.

### Uterine cytology

Uterine cytobrush sampling was performed as previously described [4,22]. Cows were restrained in a chute system, and the tail was secured to prevent movement. In animals that were agitated or anxious in the chute, caudal epidural anesthesia (3–5 mL of 2% lidocaine) was administered to reduce distress and facilitate sampling. A sterile cytobrush was inserted into a trimmed plastic sheath and mounted onto a triple-guarded metal rod. After perineal disinfection, the assembly was introduced transvaginally and guided into the uterus via rectal palpation. The cytobrush was then exposed and rotated twice to collect endometrial cells, retracted into the sheath, and the assembly was withdrawn. The cytobrush with uterine cellular material was gently rolled onto clean glass slides to prepare smears, which were then air-dried for 2–3 hours. Slides were stained using modified Wright-Giemsa stain with an automated slide stainer (Hema Tek Slide Stainer, IL, USA). A board-certified pathologist (co-author RD), blinded to treatment groups, evaluated the smears. Each slide was divided into quadrants, and a total of 400 cells per slide (100 per quadrant) were counted and classified into neutrophils, epithelial cells, macrophages, lymphocytes, and other cells (including mast cells and eosinophils).

### Statistical analyses

All statistical analyses were performed using Stata 17.0 BE software (StataCorp LLC, College Station, TX, USA) and GraphPad Prism 9. Data were assessed for normality using the Shapiro-Wilk test and Q-Q plots. Single-measure, normally distributed data (e.g., body weights) were analyzed using independent samples *t*-tests, while non-normally distributed variables (e.g., parity, body condition score) were compared using the Wilcoxon rank-sum test. Correlations between different cell proportions in uterine cytology smears were evaluated using Spearman’s correlation test. Peripheral blood cell counts measured over time were analyzed using repeated measures mixed models with an unstructured covariance matrix. Fixed effects included treatment group, sampling day, and their interaction. Uterine cell proportions were analyzed using generalized estimating equations (GEE) with a Poisson distribution, exchangeable correlation structure, and animal ID as the subject variable. The model assessed main effects (treatment group and time) and their interactions. Statistical significance was set at *P* < 0.05. Post-estimation analysis included pairwise comparisons within and between treatment groups across different time points. Granulocyte phagocytosis and oxidative burst capabilities were quantified using FLOWJO v10 software (FlowJo LLC, Ashland, OR, USA). Granulocyte populations were identified using forward and side scatter properties, with gating thresholds established using Fluorescence Minus One (FMO) controls and unstimulated samples. Within FlowJo, histogram overlays and bi-parametric plots were used to visualize shifts in fluorescence intensity. Quantitative metrics, including the percentage of positive cells and median fluorescence intensity (MFI), were calculated using the “Statistics” and “Table Editor” functions. Comparative statistical analyses of MFI and positive cell percentages between treatment and control groups were performed by GraphPad Prism 9 using unpaired t-tests.

## Results

### Study population and clinical observations

There were no significant differences between the Treatment and Control groups in mean lactation number, average daily milk yield, body condition score, or body weight at the time of enrollment (Day 0). Additionally, none of the enrolled cows exhibited clinical signs of endometritis, such as purulent vaginal discharge or the presence of intrauterine fluid on transrectal ultrasonography, throughout the study period.

### Systemic hematological responses to rG-CSF administration

**Fig 2:**
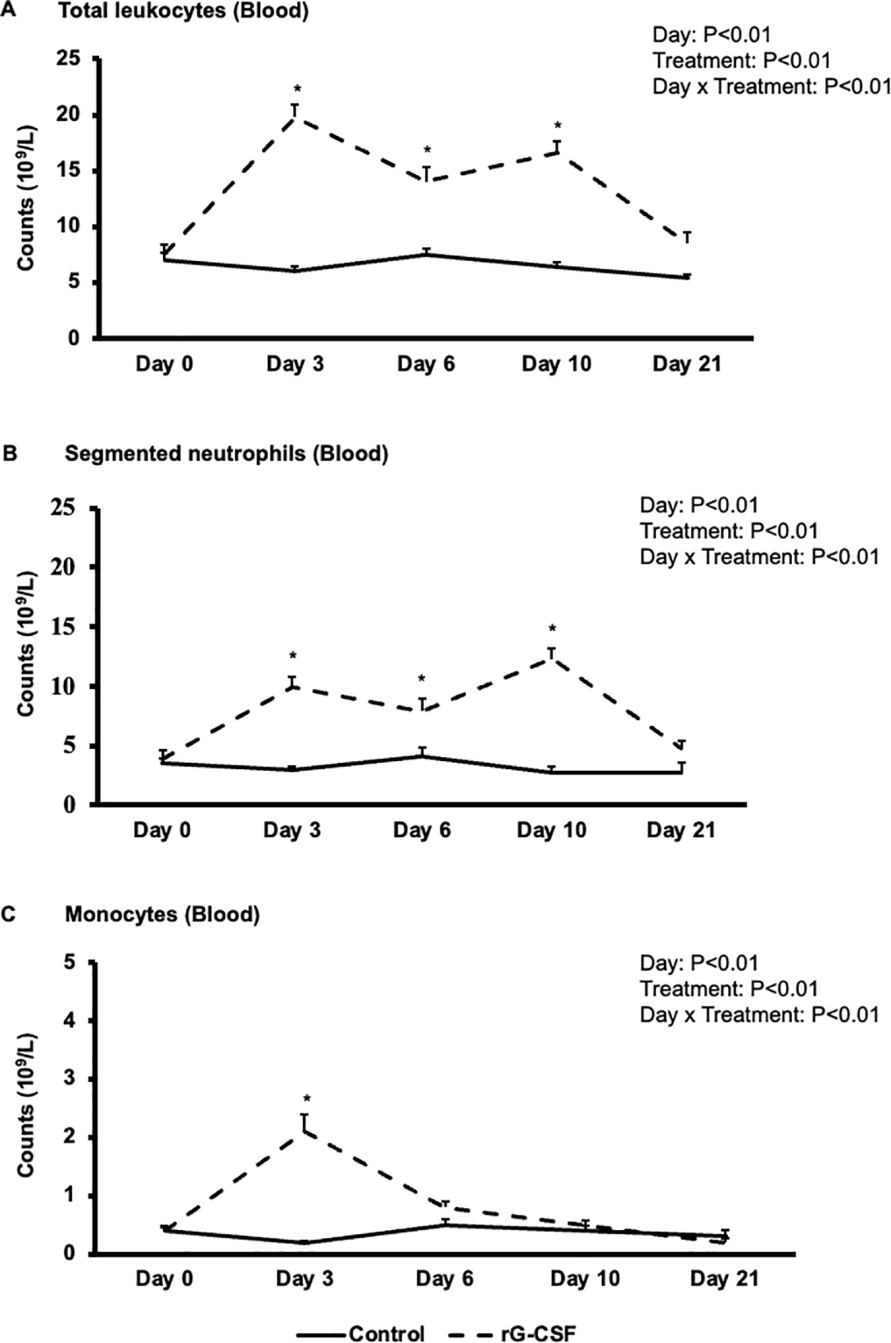
Temporal changes in total leukocyte (A), neutrophil (B), and monocyte (C) counts (Mean ± SE) in the peripheral blood of pegbovigrastim (rG-CSF, recombinant granulocyte colony-stimulating factor, n = 12) and saline-treated (Control, n = 11) postpartum cows. Blood samples were collected from postpartum cows (19-23 DIM) just prior to treatment (Day 0) and on Days 3, 6, 10, and 21 post-treatment. Significant difference (P < 0.05) between the groups on the respective sampling days are indicated by asterisks (*).

Consistent with the randomized study design, there were no significant differences in total or differential leukocyte counts in peripheral blood between the Control and rG-CSF groups prior to treatment (Day 0). Following treatment, a clear and rapid leukocytosis was observed in rG-CSF-treated cows. There was a significant effect of treatment, sampling day, and their interaction on total leukocyte, neutrophil, and monocyte counts (Treatment: P < 0.01; Day: P < 0.01; Treatment × Day: P < 0.01). Cows in the rG-CSF group exhibited a 2- to 3-fold increase (P < 0.01) in total leukocyte and segmented neutrophil counts on Days 3, 6, and 10 post-treatment compared to Control cows (Fig 2A and 2B). By Day 21, these counts had returned to levels comparable to those of the Control group.

Band and toxic neutrophils were absent in the Control group but were evident in the rG-CSF-treated cows. The highest band cell counts were observed on Day 3 (3.5 × 10⁹/L ± 0.4), followed by a decline on Days 6 and 10 (1.6 × 10⁹/L ± 0.2 and 0.7 × 10⁹/L ± 0.2, respectively), and minimal counts on Day 21 (0.03 × 10⁹/L ± 0.02) (Treatment: P < 0.01; Day: P < 0.01; Treatment × Day: P < 0.01). Toxic neutrophils were noted on Days 3 and 6 in rG-CSF-treated animals only (S1 Data). Monocyte counts in the rG-CSF group increased significantly on Day 3 (approximately 4-fold vs. Control; P < 0.01), with no significant differences observed between groups on Days 6, 10, or 21 (Fig 2C).

### Peripheral granulocyte phagocytic activity and capacity

**Fig 3:**
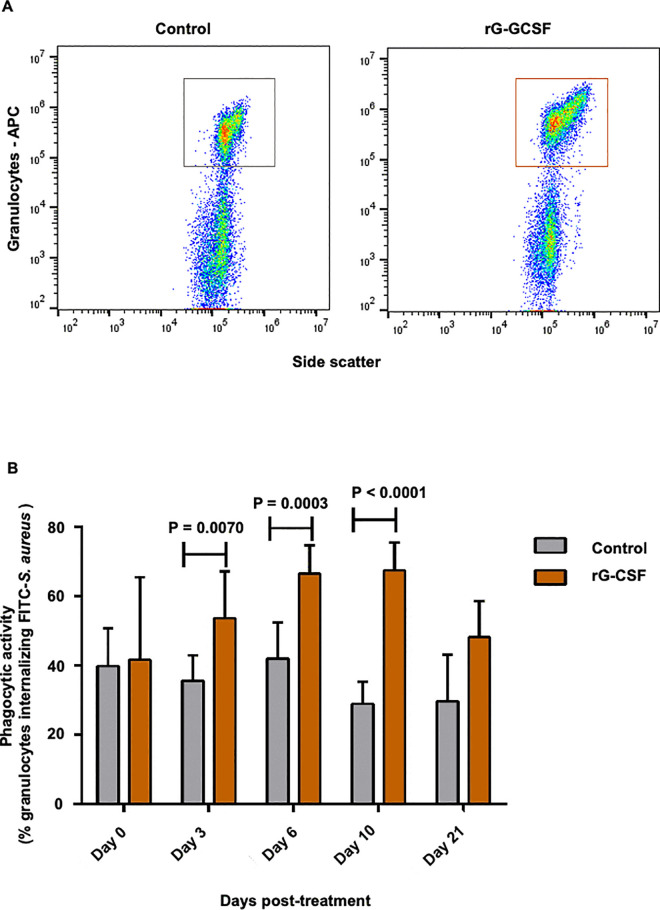
Effect of parenteral administration of pegbovigrastim on granulocyte phagocytic activity. (A) Forward (FSC) and side scatter (SSC) properties in combination with CH138A–allophycocyanin labeling (APC) were used to identify the granulocyte population; these were further gated FITC-labeled *S. aureus* internalization. (B) Comparison of granulocyte phagocytic activity between treatment groups on different days post-treatment, expressed as the proportion (%) of granulocytes positive for internalized FITC-labeled *Staphylococcus aureus*. Postpartum cows were treated with pegbovigrastim (rG-CSF, recombinant granulocyte colony-stimulating factor; n = 12) or saline (Control; n = 11). Blood samples were collected at 19–23 DIM just prior to treatment (Day 0) and on Days 3, 6, 10, and 21 post-treatment. Significant differences between treatment groups on specified sampling days are indicated by P < 0.05 in panel B.

**Fig 4:**
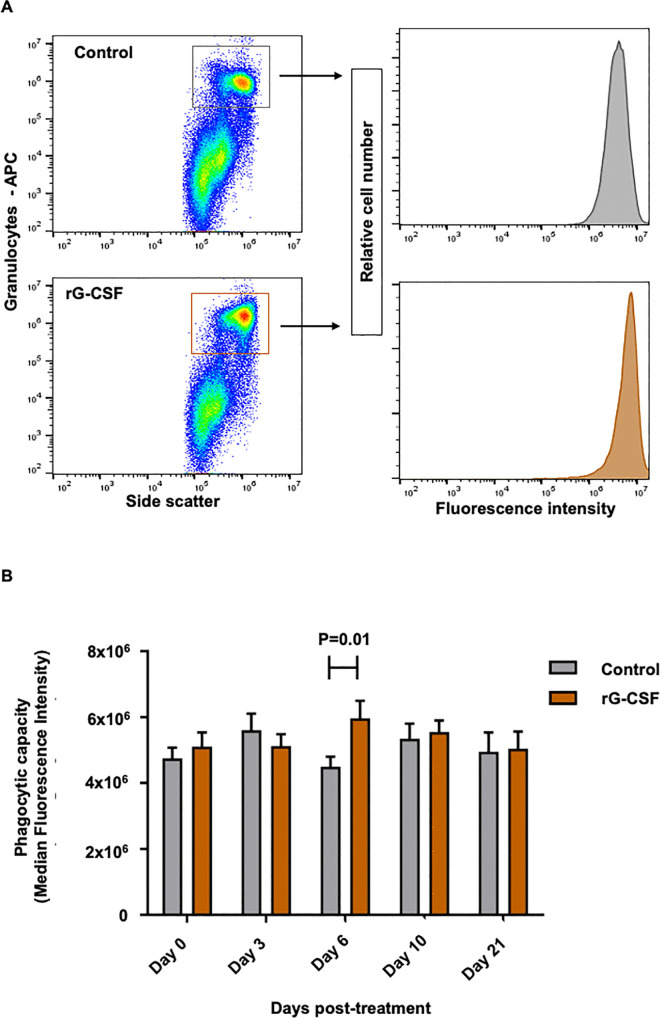
Effect of parenteral administration of pegbovigrastim on granulocyte phagocytic capacity. (A) Forward scatter (FSC) and side scatter (SSC) properties in combination with CH138A–allophycocyanin (APC) labeling were used to identify granulocyte population; these were further gated FITC-labeled *S. aureus* internalization and assessed for fluorescent intensity. (B) Comparison of granulocyte phagocytic capacity between groups on different days post-treatment, expressed as the median fluorescent intensity (MFI) of the FITC signal. This measure reflects the average number of internalized FITC-labeled *S. aureus* per cell. Postpartum cows were treated with pegbovigrastim (rG-CSF, recombinant granulocyte colony-stimulating factor, n = 12), or saline (Control, n = 11). Blood samples were collected from postpartum cows (19-23 DIM) just prior to treatment (Day 0), and then on Days 3, 6, 10, and 21 post-treatment. Significant differences between treatment groups on specified sampling days are indicated by P < 0.05 in panel B.

Phagocytic activity, defined as the proportion of granulocytes that successfully internalized FITC-labeled S. aureus, increased significantly in rG-CSF–treated animals compared with the control group on Day 3 post-treatment (P = 0.007) and exhibited an approximately two-fold increase by Day 10 (P < 0.0001; Fig 3B). Post-treatment changes in granulocyte phagocytic capacity, measured by the median fluorescence intensity (MFI) of the FITC signal, was significantly higher (~25%, P = 0.01) in rG-CSF cows compared to controls (Fig 4B). No significant differences were observed at other time points. These results suggest that a single subcutaneous administration of pegbovigrastim significantly enhances recruitment of cells into the active phagocytic pool (activity). Additionally, the treatment increases individual granulocyte efficiency in bacterial internalization (capacity). Notably, these effects follow distinct temporal patterns.

### Peripheral granulocyte oxidative burst capacity

**Fig 5:**
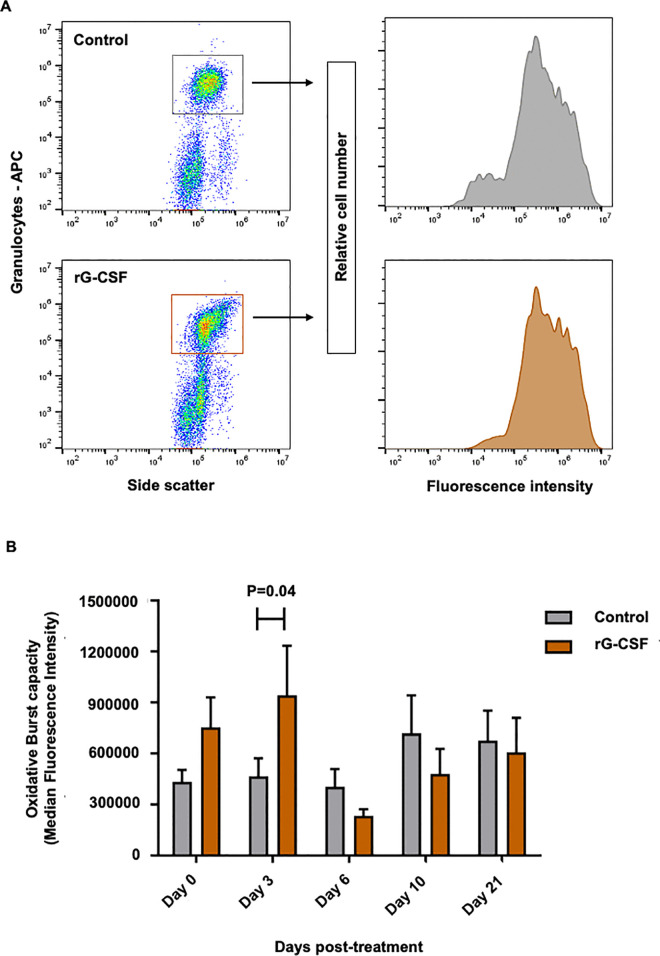
Effect of parenteral administration of pegbovigrastim on oxidative burst capacity of peripheral granulocytes. (A) Representative flow cytometry plots show cell selection based on characteristic forward scatter (FSC) and side scatter (SSC) properties in combination with CH138A–allophycocyanin labeling to identify target cell population. (B) Comparison of granulocyte oxidative burst capacity between groups on different days post-treatment, expressed as the median fluorescent intensity (MFI) of rhodamine 123 signal. Postpartum cows were treated with pegbovigrastim (rG-CSF, recombinant granulocyte colony-stimulating factor, n = 12), or saline (Control, n = 11). Significant differences between treatment groups on specified sampling days are indicated by P < 0.05 in panel B.

The oxidative burst capacity of peripheral granulocytes in rG-CSF-treated cows was significantly enhanced (~100% increase, P = 0.04, Fig 5) on Day 3 post-treatment relative to Control group cows, with no differences observed on subsequent sampling days (Fig 5B).

### Intra-uterine correlation between leukocyte and epithelial cells

**Fig 6:**
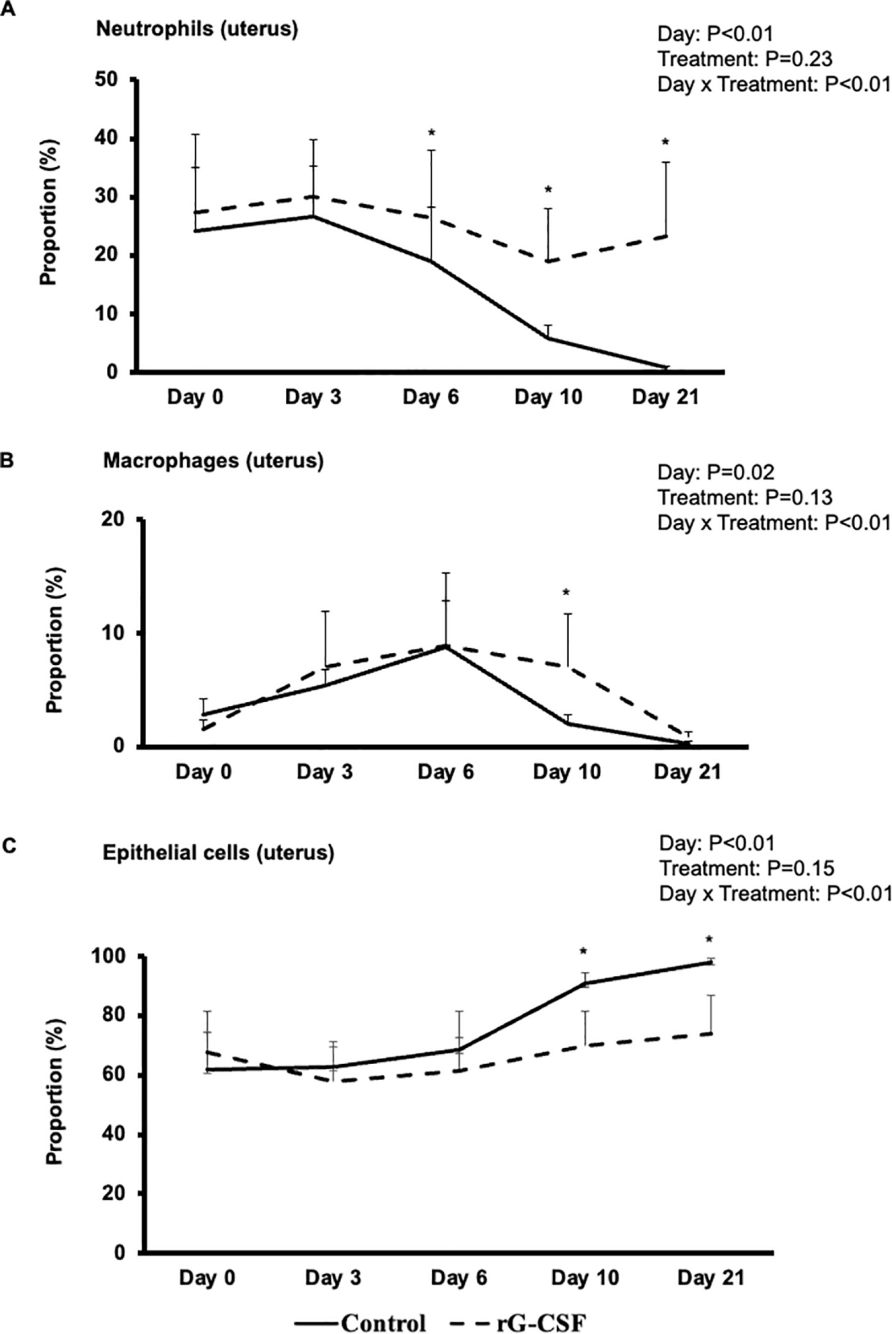
Proportions (Mean ± SE) of neutrophils (A), macrophages (B) and epithelial cells (C) in the uterus of pegbovigrastim (rG-CSF, n = 8) and saline-treated (Control, n = 7) postpartum cows. Uterine cytobrush samples were collected from postpartum cows (19-23 DIM) and cytosmears were prepared just prior to treatment (Day 0), and then from a subset of cows on Days 3, 6, 10, and 21 post-treatment. One hundred cells were counted in each quadrant of a cytosmear. Significant differences (P < 0.05) between groups on specified sampling days are indicated by asterisks (*) in the respective panels.

Following the observation of increased systemic leukocyte numbers and enhanced granulocyte function, we examined whether these changes were associated with altered leukocyte proportions within the reproductive tract. Consequently, we evaluated the temporal changes in uterine cell populations via cytosmears (Fig 6). Epithelial cell proportions showed strong negative correlation with neutrophils (Spearman’s ρ = –0.93, P < 0.001), and macrophages (Spearman’s ρ = –0.72, P < 0.001).

### Temporal changes in uterine immune cells

Neutrophil proportions (Fig 6A) in uterine cytosmears were significantly affected by sampling day (P < 0.01) and the treatment-by-time interaction (P < 0.01). In the Control group, neutrophil proportions were similar on Days 0 and 3 (P = 0.20), but declined sharply by 5, 18, and 23 percentage points on Days 6, 10, and 21, respectively (P < 0.01). In rG-CSF cows, neutrophil proportions remained stable from Days 0–6, with a moderate decrease of 4–7 percentage points on Days 10 and 21 (P < 0.01). Between groups, no differences were observed on Days 0 and 3; however, rG-CSF-treated cows had significantly higher neutrophil proportions by 6, 14, and 21 percentage points on Days 6, 10, and 21, respectively (P < 0.01).

Macrophage proportions (Fig 6B) also varied by sampling day (P = 0.02) and treatment-by-time interaction (P < 0.01). In the Control group, macrophage proportions increased significantly by 2.5 and 6 percentage points on Days 3 and 6, respectively, compared to other days (P < 0.01). In alignment with the peak peripheral monocytosis observed on Day 3, macrophage proportions in the rG-CSF group increased significantly on Days 3, 6, and 10 compared to Days 0 and 21 (P < 0.01). Between groups, macrophage proportions were comparable on Days 0, 3, 6, and 21 (P = 0.21), but were 5 percentage points higher (P < 0.01) in the rG-CSF group on Day 10. Proportions of lymphocytes, eosinophils, and mast cells in uterine cytosmears did not differ significantly between sampling days or treatment groups and are therefore not presented.

### Temporal changes in uterine epithelial cells

Epithelial cell proportions (Fig 6C) varied significantly with sampling day (P < 0.01) and the interaction between treatment and time (P < 0.01). In Control cows, epithelial cell proportions remained stable between Days 0 and 3, increased by 8% on Day 6 (P = 0.04), and rose further by 25–30% on Day 21 (P < 0.01). In contrast, rG-CSF-treated cows showed a 9% decrease in epithelial cell proportions on Day 3 compared to Day 0 (P < 0.05), with similar levels observed on Days 0, 6, and 10. However, by Day 21, epithelial cell proportions increased significantly (P < 0.03) by 9–16 percentage points. Between groups, epithelial cell proportions were similar on Days 0, 3, and 6, but were significantly higher in the Control group on Days 10 (20% higher) and 21 (24% higher) compared to the rG-CSF group.

## Discussion

This study demonstrated that a single subcutaneous injection of rG-CSF induces a multi-compartmental immune response in postpartum dairy cows between the third and sixth week postpartum. Administration of rG-CSF was associated with marked leukocytosis, characterized by neutrophilia (predominantly segmented neutrophils) and monocytosis, lasting up to ten days post-treatment. Additionally, rG-CSF enhanced phagocytic activity and capacity as well as oxidative burst capacity of granulocytes at specific time points following administration.

To the best of our knowledge, this is the first study to integrate systemic hematological profiles with longitudinal uterine cytology in postpartum dairy cows treated with rG-CSF. We observed distinct temporal patterns between peripheral blood leucocyte dynamics and changes in uterine cells populations. Specifically, rG-CSF-treated cows maintained elevated proportions of uterine neutrophils and macrophages, suggesting a temporal association between systemic leukocyte expansion and uterine leukocyte presence. The observed temporal alignment between peak systemic leukocytosis and sustained uterine leukocyte presence allowed us to reject the null hypothesis, suggesting that systemic immunomodulation may coincide with local immune shifts.

While most previous reports focused on administering rG-CSF twice, once seven days before calving and again immediately after, to reduce the incidence of mastitis [10–13], our findings demonstrate that even a single administration is associated with sustained elevations in leukocyte numbers for more than a week. This contrasted with previous studies, where leukocyte counts remained elevated for up to two weeks following the second rG-CSF dose. A subsequent study showed that a single, lower-dose rG-CSF treatment at calving also increased circulating neutrophils, band cells, and macrophages in a dose-dependent manner [13]. In both current and previous reports, increases in band neutrophils during the first week post-treatment were observed [13,23], consistent with bone marrow stimulation and release of immature neutrophils following rG-CSF [24]. Interestingly, toxic neutrophils exhibiting Döhle bodies and cytoplasmic vacuolation were identified in peripheral blood on Days 3 and 6 post-treatment. This subpopulation may reflect accelerated maturation and rapid marrow release rather than impaired cell health or function. Although toxic neutrophils have not been previously reported in cattle, similar observations have been noted in rG-CSF-treated humans [25]. In contrast to a prior study reporting lymphocytosis following rG-CSF administration [13], lymphocyte counts in the present study were not significantly altered. This discrepancy may be attributable to the smaller sample size or the single-dose protocol compared with the multiple doses regimens used in other reports.

Functionally, mature neutrophils play critical roles in pathogen elimination through phagocytosis and oxidative burst. In the current study, rG-CSF exerted distinct, time-dependent changes in granulocyte function, with enhanced oxidative burst observed on Day 3 and phagocytic activity and capacity peaking on Day 6 post-treatment. This temporal progression may reflect the multi-stage influence of rG-CSF on the myeloid lineage. The initial surge in oxidative burst on Day 3 could be related to the immediate activation of existing circulating neutrophils and the rapid release of mature cells from the bone marrow [26,27]. This notion is supported by transcriptomic evidence from an earlier study, which showed that rG-CSF upregulated genes encoding pro-inflammatory mediators and NADPH oxidase components, key drivers for the respiratory burst [28]. Conversely, the peak in phagocytosis on Day 6 may represent a new generation of neutrophils that matured entirely under the influence of rG-CSF. These cells potentially arrive with higher densities of surface receptors required for efficient pathogen engulfment [26]. This interpretation aligns with reports demonstrating transcriptional upregulation of key recognition receptors, such as *CD14* and various Fc receptors, following rG-CSF administration [29,30]. While a recent study reported increased myeloperoxidase activity (a proxy for oxidative burst) in neutrophils one week after rG-CSF administration at calving, phagocytic function was not assessed in that trial [13]. In another study involving two rG-CSF doses administered a week apart, myeloperoxidase activity rose after the first dose and remained elevated for up to five days following the second dose; however, no significant differences in neutrophil phagocytic activity were reported between treated and control cows [10]. Our findings suggested a more nuanced, biphasic response where oxidative priming likely preceded maximal phagocytic maturation. Although certain experimental models, such as specific co-culture systems, have not observed increased migration or phagocytosis [31], the concordance between our functional data and previously reported upregulated transcripts involved in pathogen recognition supports rG-CSF’s role in bolstering innate immune defense [30]. Collectively, the weight of evidence [10–13], including our study, supports rG-CSF’s role in enhancing neutrophil numbers and granulocyte functions in periparturient dairy cattle. Moreover, rG-CSF has been shown to affect monocyte and lymphocyte populations [20], although its impact on their phenotype and functional changes in peripheral circulation and the uterus warrants further investigation.

A novel and important finding of this study was the alteration in uterine cytology following a single rG-CSF administration during the third week postpartum. Although, the uterine microbial profile was not evaluated, such data would have offered valuable insight into the relationship between immune cell infiltration and microbial clearance. In saline-treated controls, the progressive decline in uterine neutrophils and a concurrent increase in epithelial cell proportions over time reflected normal uterine involution [3]. In contrast, rG-CSF-treated cows displayed a divergent uterine cytological profile, which may be associated with the systemic expansion of the peripheral leukocyte pool. Neutrophil percentages in the uterus remained elevated at Day 21 post-treatment compared with controls. This sustained elevation may reflect the systemic surge in neutrophils following rG-CSF treatment, potentially providing a continued source of cells to the uterus. This extended immune response was unexpected, particularly since no further measurements were obtained beyond Day 21. Macrophage proportions were also significantly elevated on Day 10 in treated animals. While increased neutrophil infiltration could be interpreted as cytological endometritis, it is important to note that cows with persistent uterine infections often display insufficient neutrophil responses [3]. Furthermore, rG-CSF treatment has been shown to induce uterine expression of genes related to antimicrobial peptide production [30]. Together, these findings suggest that the influx of functionally competent neutrophils and macrophages may contribute to local immune defense, although direct measures of pathogen clearance were not assessed. These observations underscore the need to optimize the timing and dosage of rG-CSF to balance effective immune activation with resolution of inflammation. Future studies should investigate various doses and treatment frequencies to better define the role of rG-CSF in the uterine immune defense and to assess its potential efficacy in preventing or treating endometritis in dairy cattle.

In conclusion, a single parenteral administration of rG-CSF in early postpartum dairy cows resulted in transient leukocytosis, neutrophilia, and monocytosis, accompanied by enhanced peripheral granulocyte function. Importantly, rG-CSF administration was also followed by an increased uterine presence of innate immune cells, suggesting that rG-CSF may modulate local uterine immunity in addition to its systemic hematological effects. While this study demonstrates an association between rG-CSF administration and uterine cell shifts, it is important to note the limitations of current study. First, the observational nature of the uterine cytology does not provide a direct mechanistic link as we did not utilize cell-tracking or chemotactic gradient analysis to confirm that peripheral cells were the same ones populating the uterus. Second, because the study was conducted on healthy postpartum cows, the clinical significance of these immunological shifts on actual disease resolution or long-term fertility remains speculative. Future large-scale clinical trials are required to determine whether these transient shifts in innate immune cells translate into improved uterine health and reproductive outcomes.

## Supporting information

S1 Data Data used for analyses of endpoints. (XLSX)
